# Retraction: Nitrogen and potassium application effects on productivity, profitability and nutrient use efficiency of irrigated wheat (*Triticum aestivum* L.)

**DOI:** 10.1371/journal.pone.0323141

**Published:** 2025-05-07

**Authors:** The PLOS One Editors

The *PLOS One* Editors retract this article [1] due to concerns about potential manipulation of the publication process. These concerns call into question the validity and provenance of the reported results. We regret that the issues were not identified prior to the article’s publication.

SS, GK, PS, and MHS did not agree with the retraction. SA and RK either did not respond directly or could not be reached.
